# Pulsed-field vs. cryoballoon vs. radiofrequency ablation: a propensity score matched comparison of one-year outcomes after pulmonary vein isolation in patients with paroxysmal atrial fibrillation

**DOI:** 10.1007/s10840-023-01651-4

**Published:** 2023-09-30

**Authors:** Jens Maurhofer, Thomas Kueffer, Antonio Madaffari, Robin Stettler, Anita Stefanova, Jens Seiler, Gregor Thalmann, Nikola Kozhuharov, Oskar Galuszka, Helge Servatius, Andreas Haeberlin, Fabian Noti, Hildegard Tanner, Laurent Roten, Tobias Reichlin

**Affiliations:** 1 Department of Cardiology, Inselspital, Bern University Hospital, University of Bern, Bern, Switzerland; 2 Sitem Center for Translational Medicine and Biomedical Entrepreneurship, University of Bern, Bern, Switzerland

**Keywords:** Pulmonary vein isolation, Paroxysmal atrial fibrillation, Pulsed-field ablation, Cryoballoon ablation, Radiofrequency ablation

## Abstract

**Background:**

Pulsed-field ablation (PFA) has shown favourable data in terms of safety and procedural efficiency for pulmonary vein isolation (PVI). We sought to compare procedural and 1-year follow-up data of patients with paroxysmal atrial fibrillation (AF) undergoing PVI using PFA, cryoballoon ablation (CBA) and radiofrequency ablation (RFA).

**Methods:**

Consecutive patients with paroxysmal AF undergoing a first PVI with PFA at our institution were included. For comparison, patients with paroxysmal AF undergoing a first PVI with CBA and RFA were selected using a 1:2:2 propensity score matching. The PFA group followed the standard 32-applications lesion-set protocol, the CBA group a time-to-effect plus 2-min strategy, and the RFA group the CLOSE protocol. Patients were followed with 7d-Holter ECGs 3, 6, and 12 months after ablation. The primary endpoint was recurrence of atrial tachyarrhythmia (ATa) following a blanking period of 3 months.

**Results:**

A total of 200 patients were included (PFA* n* = 40; CBA *n* = 80; RFA *n* = 80). Median procedure times were shortest with CBA (75 min) followed by PFA (94 min) and RFA (182 min*; p* < 0.001). Fluoroscopy dose was lowest with RFA (1.6Gycm^2^) followed by PFA (5.0Gycm^2^) and CBA (5.7Gycm^2^; *p* < 0.001). After a 1-year follow-up, freedom from ATa recurrence was 85.0% with PFA, 66.2% with CBA and 73.8% with RFA (*p* = 0.12 PFA vs. CBA; *p* = 0.27 PFA vs. RFA).

**Conclusion:**

In a propensity score matched analysis of patients with paroxysmal AF, freedom from any ATa 1 year after PVI using PFA was favourable and at least as good as for PVI with CBA or RFA.

**Graphical Abstract:**

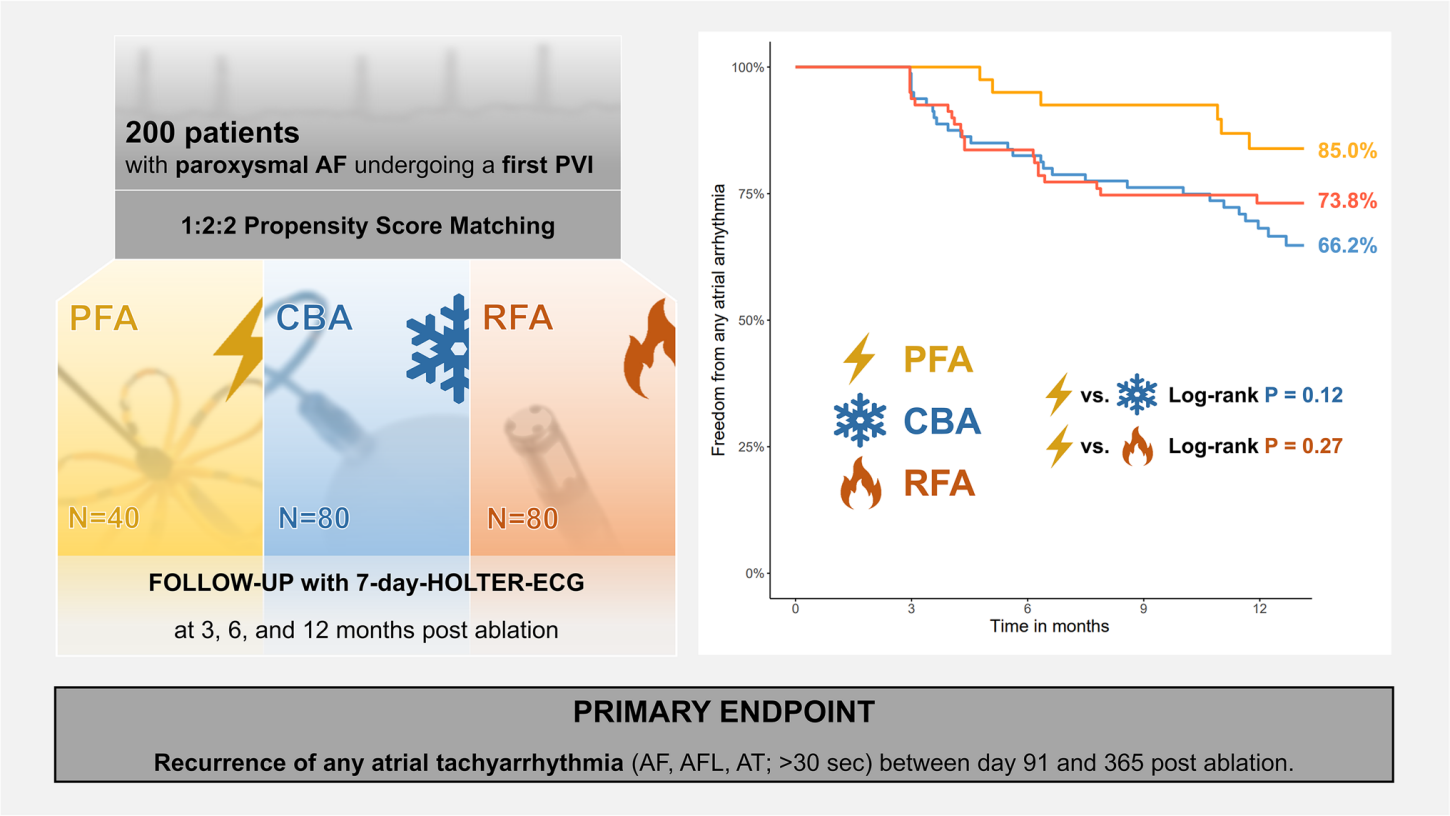

## Introduction

Atrial fibrillation (AF) is the most common arrhythmia and significantly contributes to patient morbidity and mortality [1]. Pulmonary vein isolation (PVI) using thermal ablation is a well-established treatment for paroxysmal AF [2–4]. In recent years, radiofrequency ablation (RFA) and cryoballoon ablation (CBA) protocols were constantly optimized by research and development, which reduced recurrences and improved clinical outcome [5–9]. However, recurrence of atrial tachyarrhythmia (ATa) are still common and thermal ablation may result in collateral damage such as formation of atrio-esophageal fistula or phrenic nerve palsy [10].

Since 2021, pulsed-field ablation (PFA) is commercially available in Europe for PVI in patients with AF [11–13]. PFA causes irreversible electroporation and subsequent cell death by an electrical field [14, 15]. The threshold for irreversible electroporation is tissue-specific and cardiomyocytes are most vulnerable, which allows targeted ablation of myocardial tissue without damaging adjacent tissues (e.g., esophageal wall, phrenic nerve) [16–18]. First experiences showed promising safety and acute efficacy of PFA [19], as well as favourable 12-month outcome data [20]. Studies comparing long-term effectiveness of PFA with RFA or CBA however are missing.

This study compares the 1-year efficacy of PFA with CBA and RFA in a propensity score matched cohort of patients with paroxysmal AF undergoing PVI.

## Methods

### Study population

In this prospective registry study, consecutive patients with AF undergoing a first PVI at the Inselspital, Bern University Hospital, Bern, Switzerland, were enrolled into an institutional registry (SWISS-AF-PVI Database). The registry was approved by the local ethics committee and the study was carried out in accordance with the principles of the Declaration of Helsinki. The authors had full access to the data and take full responsibility for their integrity.

**Fig. 1:**
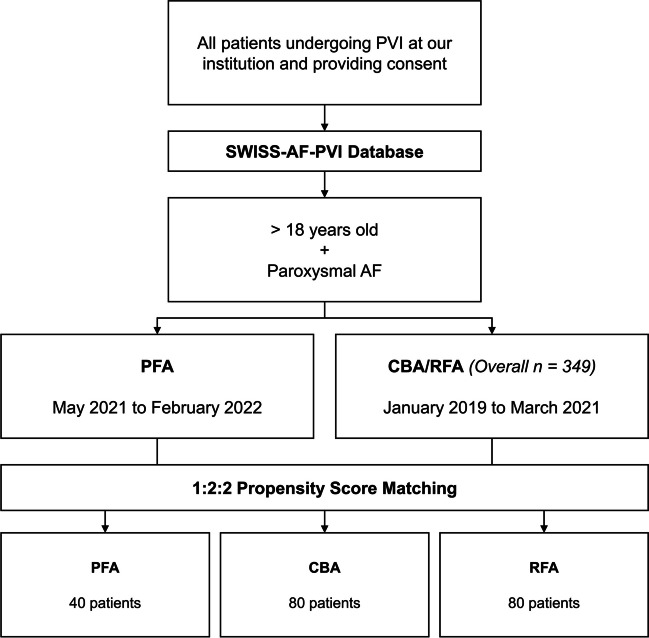
Study flowchart. *AF* atrial fibrillation, *CBA* cryoballoon ablation, *PFA* pulsed-field ablation, *PVI* pulmonary vein isolation, *RFA* radiofrequency ablation

For the purpose of this analysis, consecutive patients undergoing a first PVI for paroxysmal AF between May 2021 and February 2022 with the new PFA platform (Farapulse, Menlo Park, CA, USA) were included. For comparison, patients with paroxysmal AF were selected from the cohort of patients undergoing a first PVI with CBA and RFA between January 2019 and March 2021 (*n* = 349; 170 CBA and 179 RFA) using a 1:2:2 propensity score matching. The inclusion period for CBA and RFA patients was set before the first PFA cases to minimize selection bias. Figure 1 provides a study flowchart for illustration and further information.

### Pre-procedural examinations, sedation, and left atrial access

Prior to the procedure, patients underwent trans-esophageal echocardiography and computed tomography to exclude intracardiac thrombi and to obtain a detailed understanding of the left atrial (LA) anatomy. No patients were excluded due to anatomical particularities of the left atrium. Deep conscious sedation using midazolam, fentanyl and propofol was used, guided by a physician-led, nurse-administered protocol. No paralytics were used. A small subset of patients with a high risk of sedation complications underwent general anesthesia [21]. Left atrial access was obtained by fluoroscopy-guided transseptal puncture using a standard transseptal sheath. Heparin was administered to maintain an activated clotting time above 350 s during the procedure.

### Protocol for pulsed-field ablation

The PFA platform consists of a generator which produces the therapeutic electrical field via nanosecond-scale, high-amplitude biphasic electrical pulses (Farastar, Farapulse, Farapulse Inc., Menlo Park, California, USA). These pulses are applied via a 12-F over-the-wire multipolar single-shot ablation catheter (Farawave, Farapulse) introduced into the left atrium via a 13-F steerable sheath (Faradrive, Farapulse). The platform has been described in details previously [11]. After successful LA access, the standard transseptal sheath was replaced by the 13-F steerable Faradrive sheath. Next, the PFA catheter was introduced into the LA. A straight-tip 0.035 inch wire (Amplatzer extra-stiff, Cook group, IN, USA) or a 0.035 inch rosen-tip J-wire (InQwire, Merit Medical, UT, USA) was used to cannulate the pulmonary veins (PV). Anatomical guidance was provided by a 3D-mapping system and a mapping catheter (CARTO3 and Pentaray; Biosense Webster, Irvine, CA, USA) or by overlaying to the fluoroscopy system the outline of the 3-dimensional left atrial anatomy segmented from the CT scan. Additionally, tissue contact of the PFA catheter was confirmed visually by fluoroscopic imaging. PVI was performed with four applications in basket and four applications in flower configuration per PV as previously described to complete the standard 32-applications lesion-set [22]. Supplementary applications were delivered at the discretion of the operator in subsidiary PVs (such as a right middle PV), in case of a widespread carina, or if near-field signals remained after the standard ablation protocol. Between pairs of PFA applications, the catheter was rotated by approximately 30–40° in each configuration, in order to cover the entire circumference. Ablation was performed using a voltage of 1.9 kV until September 2021, 2.0 kV was used for successive patients. Acute PVI was verified at the end of the procedure by 3D-electroanatomical mapping (EAM) or by using the Farawave catheter in a basket configuration in all PVs with the assessment of Entrance- and Exit-Block [23].

### Protocol for cryoballoon ablation

Cryoablation was performed with a 28-mm cryoballoon catheter (Arctic Front Advance), a 20 mm circular mapping catheter (Achieve Advance), and a steerable sheath (FlexCath Advance; all Medtronic, MN, USA). After successful LA access, the standard transseptal sheath was replaced by the 12-F steerable sheath (FlexCath). The cryoballoon was then introduced and placed at the ostia of each PV to occlude the veins. In case of an effective freeze (judged by the disappearance of all local PV signals before 60 s or reaching a temperature of − 40 °C) cryoablation was continued for two additional minutes after effect (“time-to-effect plus 2-min strategy”) [24]. In the case of an ineffective freeze, the ablation was stopped and the balloon repositioned, aiming for better occlusion of the PV. Acute PVI was verified at the end of the procedure with the assessment of Entrance- and Exit-Block in all PVs using the circular mapping catheter. No adenosine test was used to detect dormant PV connection.

### Protocol for radiofrequency ablation

Radiofrequency procedures were performed using a 3D mapping system (CARTO3, Biosense Webster, Irvine, CA, USA) in combination with a CF-sensing ablation catheter (Smarttouch SF, Biosense Webster, Irvine, CA, USA) and a high-density multipolar mapping catheter (Pentaray, Biosense Webster, Irvine, CA, USA). A steerable sheath was used (Destino Reach, Oscor, Palm Harbor, FL, USA). Ablation was performed by adhering to the CLOSE protocol [5]. Power settings were at the discretion of the operator and ranged from 30 to 50 Watts. PVI was verified by 3D mapping at the end of the procedure.

### Follow-up

Follow-up visits including a 7-day-Holter electrocardiogram (ECG) were scheduled at 3, 6, and 12 months after PVI. The patients were free to choose whether they preferred to have their follow-up visits in our institution or at an external cardiologist of their choice. The primary endpoint was defined as recurrence of any atrial tachyarrhythmia ≥ 30 s (AF, AFL, or AT) between day 91 and 365 post ablation after the standard blanking period of 90 days.

### Statistical analysis

Continuous variables are presented as mean ± standard deviation or as median and interquartile range (IQR) as appropriate. Kaplan–Meier analyses with pairwise log-rank test were performed for the primary endpoint. Comparisons between independent groups were made using the chi-square method for categorical variables and using the Mann–Whitney* U* respectively Kruskal-Wallis-H test for continuous variables. Propensity score–matching was performed accounting for confounding by covariates typically affecting AF ablation outcomes: sex, age, body mass index, hypertension, diabetes mellitus, and left atrial volume index. We used 1:2:2 nearest neighbour matching with a propensity score estimate using logistic regression. Statistical analyses were made by using R 4.2.2 (R Core Team, Vienna, Austria).

## Results

### Patient characteristics

A total of 200 patients were enrolled: 40 consecutive patients with PFA PVI between May 2021 and February 2022, 80 patients with CBA PVI and 80 with RFA PVI between January 2019 and March 2021. After 1:2:2 propensity score matching, the mean of all standardized mean differences for the covariates (sex, age, hypertension, diabetes mellitus, body mass index, and Ieft atrial volume index) was below 0.1. Baseline characteristics of the propensity matched population are summarized in Table 1. The median age was 62 years (IQR 55–70), 24.5% were female and the median CHA_2_DS_2_VASc score was 2 (IQR 1–3).

**Table 1:** Baseline characteristics

	PFA	CBA	RFA	*P*-value
Baseline characteristics				
*N*	40	80	80	
Age [years]	62.6 [56.3, 68.6]	62.2 [54.7, 70.7]	62.0 [55.4, 69.7]	0.948
Age > 65 years, *n* (%)	18 (45.0)	32 (40.0)	33 (41.2)	0.870
Male sex, *n* (%)	30 (75.0)	58 (72.5)	63 (78.8)	0.653
BMI [kg/m^2^]	25.9 [23.2, 28.5]	26.2 [24.0, 28.6]	25.6 [23.6, 28.5]	0.704
Months since AF diagnosis [month]	29.0 [8.0, 83.5]	17.0 [5.0, 49.2]	11.5 [3.0, 37.2]	**0.029**
CHA_2_DS_2_VASc score, *n* (%)				0.417
0	10 (25.0)	16 (20.0)	16 (20.0)	
1	5 (12.5)	24 (30.0)	17 (21.2)	
2	12 (30.0)	18 (22.5)	22 (27.5)	
3	8 (20.0)	8 (10.0)	17 (21.2)	
4	3 (7.5)	9 (11.2)	6 (7.5)	
> 4	2 (5.0)	5 (6.2)	2 (2.5)	
Previous DCCV, *n* (%)	4 (10.0)	10 (12.5)	15 (18.8)	0.354
Previous stroke or TIA, *n* (%)	2 (5.0)	4 (5.0)	8 (10.0)	0.398
Coronary artery disease, *n* (%)	8 (20.0)	14 (17.5)	10 (12.5)	0.512
Hypertension, *n* (%)	26 (65.0)	50 (62.5)	48 (60.0)	0.862
Diabetes mellitus, *n* (%)	3 (7.5)	8 (10.0)	9 (11.2)	0.812
Left atrial diameter [mm]	42.0 [38.0, 45.8]	42.0 [38.0, 45.0]	41.0 [36.2, 45.8]	0.799
Left atrial volume index [mL/m^2^]	38.0 [33.5, 45.2]	37.3 [30.3, 46.0]	37.0 [31.3, 44.5]	0.699
Left ventricular ejection fraction [%]	60.0 [55.0, 60.0]	60.0 [55.8, 65.0]	60.0 [55.0, 60.0]	0.419

Values are presented as median [IQR] or *n* (%). *AF* atrial fibrillation, *BMI* body mass index, *CBA* cryoballoon ablation, *DCCV* direct current cardioversion, *IQR* interquartile range, *LVEF* left ventricular ejection fraction, *PFA* pulsed-field ablation, *RFA* radiofrequency ablation, *TIA* transient ischemic attack

### Procedural characteristics

Procedural characteristics of the propensity matched population are listed in Table 2. 3D-EAM was used in all patients in the RFA group and in none in the CBA group. In the PFA group, 3D-EAM was used in all 40 patients: before and after ablation in 13 patients and only after the ablation in 27. The majority of the patients receiving PVI with PFA (34/40, 85%) were treated with a 31 mm catheter. In the PFA group, the median number of applications was 32 (IQR 32–37). Besides PVI, no additional left atrial lesions were performed. Procedure times were different among groups and shortest with CBA (75 min; IQR 60–97 min) followed by PFA (94 min; IQR 80–116 min) and RFA (182 min; IQR 134–224 min; *p* < 0.001). Fluoroscopy dose was different among groups, lowest with RFA (1.6 Gycm^2^, IQR 1.0–3.2 Gycm^2^) followed by PFA (5.0 Gycm^2^, IQR 3.4–8.4 Gycm^2^) and CBA (5.7 Gycm^2^, IQR 3.0–10.2 Gycm^2^, *p* < 0.001).

**Table 2:** Procedural characteristics, periprocedural complications, and follow-up

	PFA	CBA	RFA	*P*-value
Procedural characteristics				
*N*	40	80	80	
Procedure time [min]	93.5 [79.5, 116.0]	75.0 [60.0, 97.0]	182.0 [134.2, 223.5]	** < 0.001**
Use of 3D-mapping pre-ablation, *n* (%)	13 (32.5)	0 (0.0)	80 (100.0)	** < 0.001**
Use of 3D-mapping post-ablation, *n* (%)	40 (100.0)	0 (0.0)	80 (100.0)	** < 0.001**
Fluoroscopy time [min]	25.6 [20.7, 31.0]	17.1 [12.7, 23.7]	6.7 [3.5, 12.9]	** < 0.001**
Fluoroscopy dose [Gycm^2^]	5.0 [3.4, 8.4]	5.7 [3.0, 10.2]	1.6 [1.0, 3.2]	** < 0.001**
Periprocedural complications				
Stroke, *n* (%)	0 (0.0)	0 (0.0)	0 (0.0)	> 0.999
Cardiac tamponade, *n* (%)	2 (5.0)	0 (0.0)	0 (0.0)	**0.039**
Phrenic nerve palsy, *n* (%)	0 (0.0)	0 (0.0)	0 (0.0)	> 0.999
Atrioesophageal fistula, *n* (%)	0 (0.0)	0 (0.0)	0 (0.0)	> 0.999
Follow-up				
One-year Recurrence, *n* (%)	6 (15.0)	27 (33.8)	21 (26.2)	0.091
Entity of recurrence				0.653
Paroxysmal AF	4 (66.7)	20 (74.1)	18 (85.7)	
Persistent AF	1 (16.7)	5 (18.5)	2 (9.5)	
Atypical AFL	1 (16.7)	2 (7.4)	1 (4.8)	
Redo procedure after recurrence, *n* (%)	3 (50.0)	17 (63.0)	14 (66.7)	0.887
Number of still isolated PVs, *n* (%)	8 (66.7)	36 (52.9)	29 (51.8)	0.868
Number of patients with all PVs still isolated, *n* (%)	0 (0.0)	2 (11.8)	3 (21.4)	0.681
Follow-up in patients without recurrence [months]	12.2 [11.8, 13.1]	13.1 [12.5, 13.1]	12.7 [12.0, 13.1]	**0.022**
Number of patients with a 12 months follow-up, *n* (%)	36 (90.0)	77 (96.3)	74 (92.5)	0.366

Values are presented as median [IQR] or *n* (%). *AF* atrial fibrillation, *AFL* atrial flutter, *CBA* cryoballoon ablation, *IQR* interquartile range, *PFA* pulsed-field ablation, *PV* pulmonary vein, *RFA* radiofrequency ablation

### Periprocedural complications

In the PFA group, 2/40 patients (5.0%) suffered from pericardial tamponade and required a percutaneous pericardial drainage. In both cases, perforation of the diagnostic catheter placed for backup pacing in the right ventricle was assumed causative (and confirmed during cardiac surgery in the second patient). After the second tamponade, we changed the workflow and administered 0.5 mg atropine before PFA in all patients to avoid the need for pacing from an RV catheter. No further complications occurred thereafter. No patient suffered from periprocedural stroke, persisting (> 24 h) phrenic nerve palsy, or atrioesophageal fistula in any group.

### Recurrences after pulmonary vein isolation

**Fig. 2:**
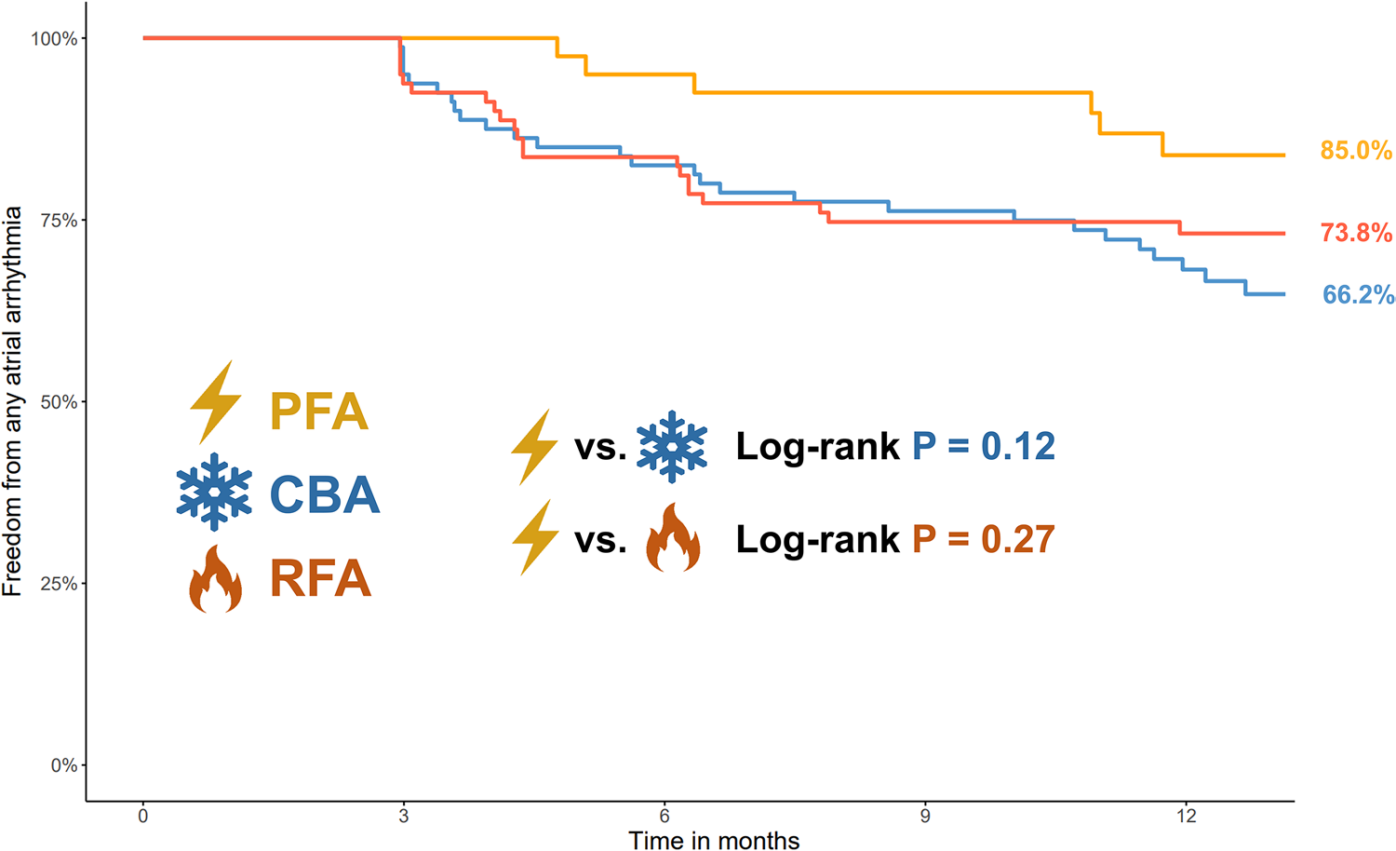
Kaplan–Meier curves of freedom from any atrial tachyarrhythmia (atrial fibrillation / atrial flutter / atrial tachycardia) after the first pulmonary vein isolation using different technologies in patients with paroxysmal atrial fibrillation**.** *CBA* cryoballoon ablation, *PVI* pulmonary vein isolation, *PFA* pulsed-field ablation, *RFA* radiofrequency ablation

Median follow-up duration of the propensity matched population was 12.8 months (IQR 12.2–13.1 months). At 12 months after the PVI, a total of 54/200 (27.0%) patients had a recurrence of any ATa. In Kaplan Meier analysis, arrhythmia-free survival after 12 months was 85.0% in the PFA group, 66.2% in the CBA group (*p* = 0.12 for comparison with PFA) and 73.8% in the RFA group (*p* = 0.27 for comparison with PFA, Fig. 2). In an exploratory analysis, there was a trend towards a lower recurrence rate in the PFA group compared to the pooled cohort of patients treated with thermal ablation strategies (PFA vs. CBA/RFA, *p* = 0.06). A higher CHA_2_DS_2_VASc score (*p* = 0.038), a higher left atrial volume index (*p* = 0.003), and a higher age (*p* = 0.019) were associated with a more frequent recurrence of atrial tachyarrhythmia after PVI.

The type of arrhythmia recurrence was paroxysmal AF in 4/6 (66.7%), persistent AF in 1/6 (16.7%), and atypical flutter in 1/6 (16.7%) patients in the PFA group. In the CBA group, it was paroxysmal AF in 20/27 (74.1%), persistent AF in 5/27 (18.5%), and atypical flutter in 2/27 (7.4%). In the RFA group, it was paroxysmal AF in 18/21 (85.7%), persistent AF in 2/21 (9.5%), and atypical flutter in 1/21 (4.8%).

3/6 patients with ATa recurrence in the PFA group, 17/27 patients in the CBA group, and 14/21 patients in the RFA group had a redo procedure. Overall, 73 of 136 PVs were durably isolated (54%). The PVI durability was 67% in the 3 PFA patients, 53% in the 17 CBA patients and 52% in the 14 RFA patients.

## Discussion

This retrospective analysis of prospectively collected data is among the first ones comparing the first commercially available PFA system to the established thermal ablation modalities, CBA and RFA. Our results provide important insights for the treatment of patients with paroxysmal AF and we report three major findings:

First, procedural characteristics were different among the groups with shortest median procedure times observed in CBA (75 min; IQR 60–97 min) followed by PFA (94 min; IQR 80–116 min) and RFA (182 min; IQR 134–224 min; *p* < 0.001). Second, safety was high with all three ablation modalities. Third and most importantly, freedom from ATa recurrence one year after ablation was 85.0% with PFA, which was at least as good as for CBA (66.2%, *p* = 0.12) and RFA (73.8%, *p* = 0.27).

### Procedural efficiency

The introduction of single shot technologies has significantly shortened procedure times for PVI [8, 25]. The PFA system used in our study allows for highly efficient PVI workflows. Median procedure times if used as a stand-alone system have been reported as low as 40 min [26]. Due to the use of 3D-mapping in all PFA patients in our study, the procedure times in the PFA group in our study were longer and also longer compared with the CBA group. After the initial learning curve and the adoption of a protocol to confirm the endpoint of PVI directly with the multipolar PFA catheter used in this study, [23] we no longer use 3D-mapping in the majority of our index PVI cases nowadays, which has shortened PFA procedures times to less than was observed in the CBA group in our study.

### Procedural safety

The tissue-specific electroporation may be beneficial in terms of safety with regards to energy-related extracardiac complications, in particular atrioesophageal fistula and phrenic nerve palsy. This observation from pre-clinical PFA studies was confirmed in MANIFEST-PF, the first large post-market registry reporting on procedural characteristics of the first 1817 patients treated with the PFA system used in this study [19]. Complications inherent to all left atrial procedures, in particular cardiac tamponade and stroke, however similarly apply to PFA systems. In our study, we had to adjust our workflow with elimination of the RV catheter initially used for back up pacing after the occurrence of cardiac tamponades due to presumed RV pacing catheter perforation in two patients. This assumption was confirmed in one patient during the surgical exploration, where an RV perforation was identified. In both patients, impressive diaphragm twitching occurred during the application of PFA, which likely lead to a drilling effect of the RV catheter. Otherwise, the data from all 200 patients enrolled in our study document the overall safety of contemporary PVI protocols regardless of the ablation modality used.

### Clinical outcomes

The recurrence rates after PFA PVI in our study were low (15.0%) and at least as favourable as observed after PVI performed with CBA (33.8%) or RFA (26.2%). Our data support the current clinical practice, which shows a trend from the conventional thermal ablation methods towards the new, non-thermal PFA technology for PVI. In line with our data, the recently presented 12-months outcome data from MANIFEST-PF showed a similarly favourable one year recurrence rate of 18.4% in patients with paroxysmal AF [20]. In this study, the follow-up strategies however were very heterogeneous between centres. Two recent international multicentre studies assessing outcomes after PVI using 2 other novel PFA devices in patients with paroxysmal AF reported somewhat higher one-year recurrence rates of 29.1% [27] and 30.5% [28]. Comparison of outcomes after PVI across studies however is challenging due to differences in study design. In particular, the monitoring strategy used to assess AF recurrence after PVI has a significant impact on the rate of recurrence that will be detected [29]. Because both studies were designed for regulatory approval, a rigorous monitoring strategy with weekly and symptomatic transtelephonic ECG monitoring in addition to regular 24-h Holter ECGs was used, which explains the higher number of detected recurrences in these 2 studies [27, 28]. It is a strength of our study that with our single-centre design, a uniform monitoring strategy using 7d-Holter-ECGs after 3, 6, and 12 months was uniformly used in all three groups, which should have largely eliminated the potential effect that can be introduced by monitoring differences. Prospective randomized studies are needed to corroborate our initial experience. Currently, 3 prospective randomized-controlled trials (ADVENT (NCT04612244), SINGLE SHOT CHAMPION (NCT05534581), BEAT-AF (NCT05159492)) are studying the efficacy of the PFA system used in our study compared to CBA and RFA and will allow to draw firmer conclusions.

An additional outcome of interest in patients with paroxysmal AF is progression from paroxysmal to persistent AF. Longterm-FU from the EARLY-AF-study as well as from the ATTEST study showed superiority of catheter ablation over medical management to prevent progression to persistent AF [9, 30]. A recent study reported a frequent occurrence of roof-dependent macro-reentrant tachycardias following PVI with PFA linked to an excessive lesion set applied to the posterior wall during the index PFA PVI [31] . In our study, the most common recurrent arrhythmia was paroxysmal AF in all three groups (PFA 66.7%; CBA 74.1%; RFA 85.7%) and the progression to persistent AF and the occurrence of atypical flutter was not different across the three technologies. Larger sample sizes will however be needed to assess for potential differences between PFA and thermal ablation technologies in that regard.

## Limitations

Our findings have to be interpreted in the light of several limitations. First, our study is limited by its non-randomized design and sample size. However, propensity score matching was performed to account for confounders typically affecting AF ablation outcomes, including sex, age, body mass index, hypertension, diabetes mellitus, and left atrial volume. Second, we incorporated the complete learning curve of the first patients treated with PFA in our institution in our analysis. Third, all patients in our study had paroxysmal AF. Our findings therefore cannot be extended to patients with non-paroxysmal AF. Similar studies will be needed to assess the value of PVI with PFA also for the important group of patients with persistent AF.

## Conclusions

In a propensity score matched analysis of patients with paroxysmal AF, freedom from any atrial tachyarrhythmia one year after PVI using PFA was favourable and at least as good as for PVI performed with CBA or RFA.

## Data Availability

The data that support the findings of this study are available from the corresponding author upon reasonable request.

## Abbreviations

AAD: Antiarrhythmic drug
AF: Atrial fibrillation
AFL: Atrial flutter
AT: Atrial tachycardia
ATa: Atrial tachyarrhythmia
CBA: Cryoballoon ablation
EAM: Electroanatomical mapping
IQR: Interquartile range
LA: Left atrial/atrium
PFA: Pulsed-field-ablation
PV: Pulmonary vein
PVI: Pulmonary vein isolation
RFA: Radiofrequency ablation

## References

[1] Chung MK, Refaat M, Shen WK, Kutyifa V, Cha YM, Di Biase L (2020). Atrial fibrillation: JACC Council Perspectives. J Am Coll Cardiol.

[2] Packer DL, Kowal RC, Wheelan KR, Irwin JM, Champagne J, Guerra PG (2013). Cryoballoon ablation of pulmonary veins for paroxysmal atrial fibrillation: first results of the North American Arctic Front (STOP AF) pivotal trial. J Am Coll Cardiol.

[3] Wilber DJ, Pappone C, Neuzil P, De Paola A, Marchlinski F, Natale A (2010). Comparison of antiarrhythmic drug therapy and radiofrequency catheter ablation in patients with paroxysmal atrial fibrillation: a randomized controlled trial. JAMA.

[4] Jaïs P, Cauchemez B, Macle L, Daoud E, Khairy P, Subbiah R (2008). Catheter ablation versus antiarrhythmic drugs for atrial fibrillation: the A4 study. Circulation.

[5] Taghji P, El Haddad M, Phlips T, Wolf M, Knecht S, Vandekerckhove Y (2018). Evaluation of a strategy aiming to enclose the pulmonary veins with contiguous and optimized radiofrequency lesions in paroxysmal atrial fibrillation: a pilot study. JACC Clin Electrophysiol.

[6] Natale A, Reddy VY, Monir G, Wilber DJ, Lindsay BD, McElderry HT (2014). Paroxysmal AF catheter ablation with a contact force sensing catheter: results of the prospective, multicenter SMART-AF trial. J Am Coll Cardiol.

[7] Ferrero-de-Loma-Osorio Á, García-Fernández A, Castillo-Castillo J, Izquierdo-de-Francisco M, Ibáñez-Críado A, Moreno-Arribas J (2017). Time-to-effect-based dosing strategy for cryoballoon ablation in patients with paroxysmal atrial fibrillation: results of the plusONE Multicenter Randomized Controlled Noninferiority Trial. Circ Arrhythm Electrophysiol.

[8] Andrade JG, Champagne J, Dubuc M, Deyell MW, Verma A, Macle L (2019). Cryoballoon or radiofrequency ablation for atrial fibrillation assessed by continuous monitoring: a randomized clinical trial. Circulation.

[9] Andrade JG, Deyell MW, Macle L, Wells GA, Bennett M, Essebag V (2023). Progression of atrial fibrillation after cryoablation or drug therapy. N Engl J Med.

[10] Natale A, Mohanty S, Goldstein L, Gomez T, Hunter TD (2021). Real-world safety of catheter ablation for atrial fibrillation with contact force or cryoballoon ablation. J Interv Card Electrophysiol.

[11] Reddy VY, Neuzil P, Koruth JS, Petru J, Funosako M, Cochet H (2019). Pulsed field ablation for pulmonary vein isolation in atrial fibrillation. J Am Coll Cardiol.

[12] Reddy VY, Anic A, Koruth J, Petru J, Funasako M, Minami K (2020). Pulsed field ablation in patients with persistent atrial fibrillation. J Am Coll Cardiol.

[13] Reddy VY, Dukkipati SR, Neuzil P, Anic A, Petru J, Funasako M (2021). Pulsed field ablation of paroxysmal atrial fibrillation: 1-year outcomes of IMPULSE, PEFCAT, and PEFCAT II. JACC Clin Electrophysiol.

[14] Davalos RV, Mir ILM, Rubinsky B (2005). Tissue ablation with irreversible electroporation. Ann Biomed Eng.

[15] Kotnik T, Kramar P, Pucihar G, Miklavcic D, Tarek M (2012). Cell membrane electroporation- Part 1: The phenomenon. IEEE Electr Insul Mag.

[16] Wittkampf FHM, van Es R, Neven K (2018). Electroporation and its relevance for cardiac catheter ablation. JACC Clin Electrophysiol.

[17] Bradley CJ, Haines DE (2020). Pulsed field ablation for pulmonary vein isolation in the treatment of atrial fibrillation. J Cardiovasc Electrophysiol.

[18] van Driel VJHM, Neven K, van Wessel H, Vink A, Doevendans PAFM, Wittkampf FHM (2015). Low vulnerability of the right phrenic nerve to electroporation ablation. Heart Rhythm.

[19] Ekanem E, Reddy VY, Schmidt B, Reichlin T, Neven K, Metzner A (2022). Multi-national survey on the methods, efficacy, and safety on the post-approval clinical use of pulsed field ablation (MANIFEST-PF). Europace.

[20] Turagam M, Neuzil P, Schmidt B, Reichlin T, Neven K, Metzner A, et al. Safety and effectiveness of pulsed field ablation to treat atrial fibrillation: one-year outcomes from the MANIFEST-PF Registry. Circulation. 2023;148:35–46. 10.1161/CIRCULATIONAHA.123.064959 37199171

[21] Servatius H, Küffer T, Baldinger SH, Asatryan B, Seiler J, Tanner H (2022). Dexmedetomidine versus propofol for operator-directed nurse-administered procedural sedation during catheter ablation of atrial fibrillation: A randomized controlled study. Heart Rhythm.

[22] Reddy VY, Koruth J, Jais P, Petru J, Timko F, Skalsky I (2018). Ablation of atrial fibrillation with pulsed electric fields: an ultra-rapid, tissue-selective modality for cardiac ablation. JACC Clin Electrophysiol.

[23] Kueffer T, Baldinger SH, Servatius H, Madaffari A, Seiler J, Mühl A (2022). Validation of a multipolar pulsed-field ablation catheter for endpoint assessment in pulmonary vein isolation procedures. Europace.

[24] Aryana A, Kenigsberg DN, Kowalski M, Koo CH, Lim HW, O’Neill PG (2017). Verification of a novel atrial fibrillation cryoablation dosing algorithm guided by time-to-pulmonary vein isolation: results from the Cryo-DOSING Study (Cryoballoon-ablation DOSING Based on the Assessment of Time-to-Effect and Pulmonary Vein Isolation Guidance). Heart Rhythm.

[25] Kuck KH, Brugada J, Fürnkranz A, Metzner A, Ouyang F, Chun KRJ (2016). Cryoballoon or radiofrequency ablation for paroxysmal atrial fibrillation. N Engl J Med.

[26] Schmidt B, Bordignon S, Tohoku S, Chen S, Bologna F, Urbanek L (2022). 5S Study: safe and simple single shot pulmonary vein isolation with pulsed field ablation using sedation. Circ Arrhythm Electrophysiol.

[27] Duytschaever M, De Potter T, Grimaldi M, Anic A, Vijgen J, Neuzil P (2023). Paroxysmal atrial fibrillation ablation using a novel variable-loop biphasic pulsed field ablation catheter integrated with a 3-dimensional mapping system: 1-year outcomes of the multicenter inspIRE Study. Circ Arrhythm Electrophysiol.

[28] Verma A, Haines DE, Boersma LV, Sood N, Natale A, Marchlinski FE, et al. Pulsed Field ablation for the treatment of atrial fibrillation: PULSED AF Pivotal Trial. Circulation. 2023 Mar 6;147:00–00. [Epub ahead of print]. 10.1161/CIRCULATIONAHA.123.063988 PMC10158608 36877118

[29] Aguilar M, Macle L, Deyell MW, Yao R, Hawkins NM, Khairy P (2022). Influence of monitoring strategy on assessment of ablation success and postablation atrial fibrillation burden assessment: implications for practice and clinical trial design. Circulation.

[30] Kuck KH, Lebedev DS, Mikhaylov EN, Romanov A, Gellér L, Kalējs O (2021). Catheter ablation or medical therapy to delay progression of atrial fibrillation: the randomized controlled atrial fibrillation progression trial (ATTEST). Europace.

[31] Tohoku S, Chun KRJ, Bordignon S, Chen S, Schaack D, Urbanek L (2023). Findings from repeat ablation using high-density mapping after pulmonary vein isolation with pulsed field ablation. Europace.

